# Role of miRNA-155 in the regulation of osteoclast differentiation mediated by MITF in stage III/IV periodontitis: a case-control study

**DOI:** 10.1186/s43141-022-00441-1

**Published:** 2022-12-02

**Authors:** Sowmya Reddy Nandipati, Devapriya Appukuttan, Sangeetha Subramanian, P. S. G. Prakash

**Affiliations:** Department of Periodontics, SRM Dental College and Hospital, Barathi Salai, Ramapuram, Chennai 600089 India

**Keywords:** microRNA, MITF, miRNA-155, Osteoclastogenesis, Periodontitis, qRT-PCR

## Abstract

**Background:**

Monocyte-macrophage lineage cells are committed towards osteoclast differentiation in vitro by the downregulation of microphthalmia-induced transcription factor (MITF) by miRNA-155. Therefore, we aimed to evaluate miRNA-155 expression and explore the regulation of MITF by miRNA-155 during osteoclastogenesis in periodontitis.

**Materials and methods:**

Ninety-eight subjects were recruited and categorized into the following: group I (cases)—systemically healthy with localized stage III/IV periodontitis (*N* = 49) and group II (controls)—systemically and periodontally healthy (*N* = 49). Gingival tissue samples were procured and qRT-PCR analysis was carried out for relative gene expression.

**Results:**

The mean ΔCT of miRNA-155 expression was −1.04 ± 2.26 and −0.01 ± 1.4 respectively for groups I and II. There was a statistically significant difference in the miRNA-155 expression (*P* ≤ 0.01) between the groups. The mean ΔCT of MITF expression for groups I and II was 4.15± 2.16 and 3.51± 1.57 respectively with no significant difference (*P* > 0.01) between the groups. In the periodontitis group, miRNA-155 expression increased by fivefolds (*P* ≤ 0.01) whereas MITF expression showed no significant difference in the fold change between the groups (*P* > 0.01). The site-specific clinical parameters showed a statistically significant strong negative and positive correlation with the ΔCT and fold change values of miRNA-155 respectively in the total 98 samples (*P* < 0.01). miRNA-155 was able to discriminate between periodontal health and disease with a diagnostic accuracy of 96.9% (95%CI: 91.38–98.95) and the AUC was 0.98 (95%CI: 0.97–1.0, SE = 0.008, *P* < 0.001) in ROC analysis with a sensitivity of 93.8% (95%CI: 83.48–97.9) and specificity of 100% (95%CI: 92.73–100).

**Conclusions:**

miRNA-155 was dysregulated and upregulated by fivefolds in periodontal disease. It can be used as a potential biomarker to discriminate between periodontal health and disease. No difference in the MITF gene expression was demonstrated between periodontal health and disease. The result suggested that miRNA-155 does not affect the expression of MITF gene in the process of osteoclastogenesis in localized stage III/IV periodontitis within this study design and limitations.

**Supplementary Information:**

The online version contains supplementary material available at 10.1186/s43141-022-00441-1.

## Background

MicroRNAs are non-coding, short, single-stranded RNAs with approximately 18–22 nucleotides that use RNA interference mechanisms to regulate gene expression at the post-transcriptional stage [1]. They regulate a wide range of biological processes, including bone formation and maintenance. Osteoclastogenesis, a complex process in the bone microenvironment, is also regulated by microRNAs via direct regulation of osteoclast activity, signaling pathways, or negative feedback loops.

Macrophage colony-stimulating factor (M-CSF) expressed by osteoblasts and their precursors bind to M-CSF receptor c-fms on the surface of osteoclast precursor cells, resulting in auto and transphosphorylation of tyrosine residues within the cytoplasmic tail end of c-fms, which activates the Erk and PI3k/Akt pathways, favoring osteoclast precursor proliferation and survival [2]. In addition, microphthalmia-induced transcription factor (MITF) is activated, which binds to B-cell lymphoma 2(Bcl2) and inhibits apoptosis [3]. The erythroblast transformation-specific (ETS) domain transcription factor PU.1 in collaboration with MITF and M-CSF mediates early and late differentiation towards the osteoclast lineage [4]. A number of miRNAs, including miR-5110, miR-340, miR-675, miR-137, miR-101, miR-96, miR-218, miR-124, miR-1276, and miR-155, can downregulate or upregulate MITF [5–9]. In humans, the MITF gene is located on the short arm of chromosome 3, and its cytogenetic location is 3p13.

miR-155 is a multifunctional miRNA that is encoded by the BIC gene on chromosome 21 and plays an important role in immune and inflammatory responses. It is coupled to T cell, B cell, and dendritic cell differentiation and activation. In hypoxic conditions, miR-155 activates autophagy by targeting the mTOR pathway, which inhibits cell cycle and proliferation, resulting in periodontal tissue destruction [10]. In vitro studies have shown that binding of LPS/TLRs and TNF-α/TNFR activates NF-kB, which increases the expression of miR-155 and activates proinflammatory cytokine genes downstream [11]. Furthermore, TLR responsiveness to leukotriene B4 is increased by miR-155 by inhibiting SOCS-1, a negative regulator, and increasing the expression of proinflammatory cytokines [9]. In vitro studies have shown that IFN-β and TGF-β inhibit the differentiation of osteoclast by inducing miR-155 that represses MITF expression in macrophages and favors the commitment of these cells towards osteoclast differentiation.

Based on the currently available scientific literature, the expression pattern of miR-155 in periodontitis is contentious and needs further exploration. Further, miRNA-mediated mechanisms are emerging as an important pathological factor in bone-related diseases particularly, the negative regulation of MITF by miR-155. Till date, no studies have evaluated the role of miR-155 in regulating osteoclastogenesis mediated via MITF in periodontitis. Therefore, the present study aimed to evaluate the expression of miRNA-155 in localized stage III/IV periodontitis and to determine the relationship between miR-155 and MITF that will further enhance our understanding on epigenetic mechanisms underlying alveolar bone resorption in periodontal disease pathogenesis.

## Methods

### Ethical clearance and sample size calculation

The subjects were recruited from the Department of Periodontics and Oral Implantology at SRM Dental College, Chennai, outpatient clinic. Patients were enrolled from January 2020 to June 2021 after the Institutional Scientific and Ethical Review Board gave its approval for the research (IEC approval: SRMDC / IRB / 2019 / MDS / No. 506). Specific inclusion and exclusion criteria were used to determine recruitment, and gingival samples were collected for analysis. All participants provided written informed consent following an explanation of the study's objectives, protocol, risks, and potential benefits to them.

### Study population, eligibility criteria, and clinical parameters recorded

Sample size was calculated using G Power software based on the article by Radović et al. [12]. The mean values with effect size of 0.8 were used for analysis. To achieve 90% power with 1% α error, a sample size of 98 was required. The statistical significance was set to *P*≤ 0.01 (*α*=1%). The study subjects were assigned into 2 groups: group 1 (test): *N* = 49, systemically healthy patients with localized stage III/IV periodontitis, and group 2 (control): *N* = 49, systemically and periodontally healthy subjects with no clinical attachment loss.

#### Inclusion criteria

Group I: Age ≥ 18years, patients with untreated stage III or stage IV periodontitis (according to 2017 EPF/AAP Classification), Gingival Index ≥ 1, periodontal probing depth ≥ 6mm, clinical attachment loss ≥ 3mm

Group II: Age ≥ 18years, systemically healthy, probing depth ≤ 3mm, no clinical attachment loss, Gingival Index score <1, presence of at least 20 natural teeth excluding 3rd molars

#### Exclusion criteria

Patients with any systemic or autoimmune diseases; patients under medication with immunosuppressants, antibiotics, and anti-inflammatory drugs in the last 6 months; pregnant or lactating mothers; smokers; and patients with previous history of periodontal therapy.

The subjects underwent routine periodontal examination and the clinical parameters were recorded by a single calibrated examiner to avoid bias. The site-specific clinical parameters like Probing Pocket Depth (in mm), Clinical Attachment Level (in mm), Plaque Index (PI) [Silness and Loe, 1964], and Gingival Index (GI) [Loe and Silness,1963] were recorded.

### Gingival tissue sample collection

During the study period, there was a complete lockdown declared from March 2020 till Aug 2020, second lockdown was from mid-April 21 till mid-June 21, the institution started functioning post-lockdown with appropriate COVID protection protocols, and the patients were treated for emergency procedures like extractions after they were declared negative in RT-PCR analysis. Further, orthodontic treatment also resumed following appropriate COVID protection protocol and negative RT-PCR report. Gingival tissue samples were collected for group I from hopeless prognosis tooth diagnosed with stage III/IV periodontitis indicated for extraction and for group II from patients scheduled for extraction of periodontally healthy premolars for orthodontic purposes and from those undergoing crown lengthening procedure in a non-inflamed periodontium. Local infiltration was given using 2% lignocaine with 1:80,000 adrenaline and then using 15c blade the gingival tissues were excised using intracrevicular and inverse bevel incisions on the facial/lingual/interproximal gingiva to obtain a single tissue biopsy measuring 3mm × 5mm. The tissues were placed in a 2-ml Eppendorf tube containing RNA later solution and stored at −20°C.

### Quantitative real-time PCR analysis

miRNA isolation was performed with the help of a miRNA isolation kit (Invitrogen mirVana^TM^, Thermo Fisher Scientific, USA) and phenol. The miRNA DNA synthesis kit (TaqMan™ MicroRNA Reverse Transcription Kit Catalog number: 4366597) was used for preparing the cDNA template to evaluate miRNA expression levels using real time-PCR (Rotor Gene Q, Qiagen, Venlo, Netherlands). miRNA expression master mix (MM) was prepared using TaqMan Advanced miRNA assay according to the manufacturer’s instructions.

The primers used for qRT-PCR are as follows: miRNA.-155: 5′CUGUUAAUGCUAAUGUGUAGGGGUUUUGC3′ (forward), 3′GACAAUUACGAUUAUACAUCCUCAGAACU 5′ (reverse); miRNA-361-5p: 5′ UUAUCAGAAUCUCCAGGGGUAC 3′ (forward) (endogenous control) (Taqman Micro Assay, Thermo Fisher Scientific, USA catalog number:4427975). The RNA isolation procedure for the MITF gene was carried using the RNAiso plus Reagent (TRIZOL) and then cDNA was prepared using High Capacity Reverse Transcription Kit (TaqMan™ High-Capacity cDNA Reverse Transcription Kit, Catalog no:4368814) following manufacturer’s protocol. Gene expression master mix was prepared using SYBR Green dye (Sigma Aldrich KAPA SYBR® FAST Universal 2X qPCR Master Mix, KK4600) for MITF and GAPDH gene expression (endogenous control). The primer pairs used for MITF were 5′-ACTTTCCCTTATCCCATCCACC-3′ (forward), 5′-TGAGATCCAGAGTTGTCGTACA-3′ (reverse); for GAPDH, 5′-TGCACCACCAACTGCTTA-3′ (forward), 5′-GATGCAGGGATGATGTTC-3′ (reverse) primer sets were used. The RT-PCR was carried out for all the cDNA-converted samples in duplicates.

The relative expression of miRNA-155 and MITF was evaluated using Livak method and reported as fold change normalized to the endogenous control gene as follows:

- ❖ ΔCT = CT of the target DNA − CT of the housekeeping gene.
- ❖ ΔΔCT = ΔCT value of the sample for a particular target − average ΔCT value of the control group for the same target.
- ❖ The relative expression (Fold Change) of miRNA = 2^−ΔΔCT^.

## Statistical analysis

To analyze the data, SPSS (IBM SPSS Statistics for Windows, Version 26.0, Armonk, NY: IBM Corp. Released 2019) was used. Significance level was fixed as 1% (*α* = 0.01). The Normality test revealed that the clinical variables were non-normally distributed whereas the other variables were normally distributed. Therefore, both non-parametric and parametric tests were applied. The descriptive data was expressed in the form of mean ± standard deviation and first-quartile, median, and third-quartile values. Age and gender distribution between the groups were compared using independent t test and chi square test respectively. Independent *t*-test was applied for intergroup comparison of standardized ΔCT and fold change. Mann-Whitney *U* test was applied for comparison of site-specific clinical parameters between the groups. Spearman rank correlations are calculated to assess the linear relationship between variables. Receiver operating curve (ROC) analysis was plotted based on the miRNA-155 and MITF standardized CT values to evaluate their ability to be used as a diagnostic marker for periodontitis.

## Results

### Characteristics of the participants and the comparison of clinical parameters between groups I and II (Table 1)

It was observed that 32 males (65.3%) and 17 females (34.7%) were present in group I and 26 males (53.1%) and 23 females (46.9%) were present in group II. No significant difference was observed in the gender distribution between groups (*χ*^2^=1.52, *P*>0.01). Likewise, no significant difference was observed between the groups based on the mean age of the participants (*P* = 0.14, ≥ 0.01) (Table 1).

**Table 1:** Mean age and gender distribution of the study participants

		Group I	Group II	Statistical test *P* value
Age (in years)^ϯ^		44.75±2.66	44.24 ± 2.00	*P* >0.01, NS
Gender^Ϯ^	Male (*n*, %)	32 (65.3%)	26 (53.1%)	*P*>0.01, NS
	Female (*n*, %)	17 (34.7%)	23 (46.9%)	

^Ϯ^Chi-square test, ^ϯ^independent *t* test, *P*>0.01, *NS*- not statistically significant

Individuals diagnosed with periodontitis had higher site specific mean PPD (in mm), mean CAL (in mm), mean PI, and mean GI scores than those with healthy periodontium. Intergroup comparison of clinical parameters demonstrated a statistically significant difference (*P*<0.001) (Table 2).

**Table 2:** Site-specific clinical parameters evaluated in groups I and II

		Group I	Group II	Statistical test *P* value
SS-PPD^$^ (mm)	Mean±SD	8.2±1.3	1.39±0.49	*P* <0.01^**^
	Median (Q1–Q3)	7.80 (7.5–8.6)	1.30 (1.0–2.0)	
SS-CAL^$^ (mm)	Mean±SD	10.87±1.82	0	*P* <0.01^**^
	Median (Q1–Q3)	10.30 (9.6–11.3)	0	
SS-PI^$^	Mean±SD	1.86 ± 0.26	0.65±0.18	*P* <0.01^**^
	Median (Q1–Q3)	2.0 (1.75–2.0)	0.75 (0.75–0.75)	
SS-GI^$^	Mean±SD	1.24±0.28	0.17±0.19	*P* <0.01^**^
	Median (Q1–Q3)	1.0 (1.0–1.0)	0.25 (0–0.25)	

*SS-PPD* site-specific mean probing pocket depth, *SS-CAL* site-specific mean clinical attachment loss, *SS-PI* site-specific Plaque index score, *SS-GI* site-specific Gingival Index score, *Q1–Q3* lower quartile to upper quartile

^$^Mann-Whitney test

^**^P value ≤0.01, statistically significant

### Comparison of the miRNA-155 and MITF gene expression between groups I and II

**Fig. 1:**
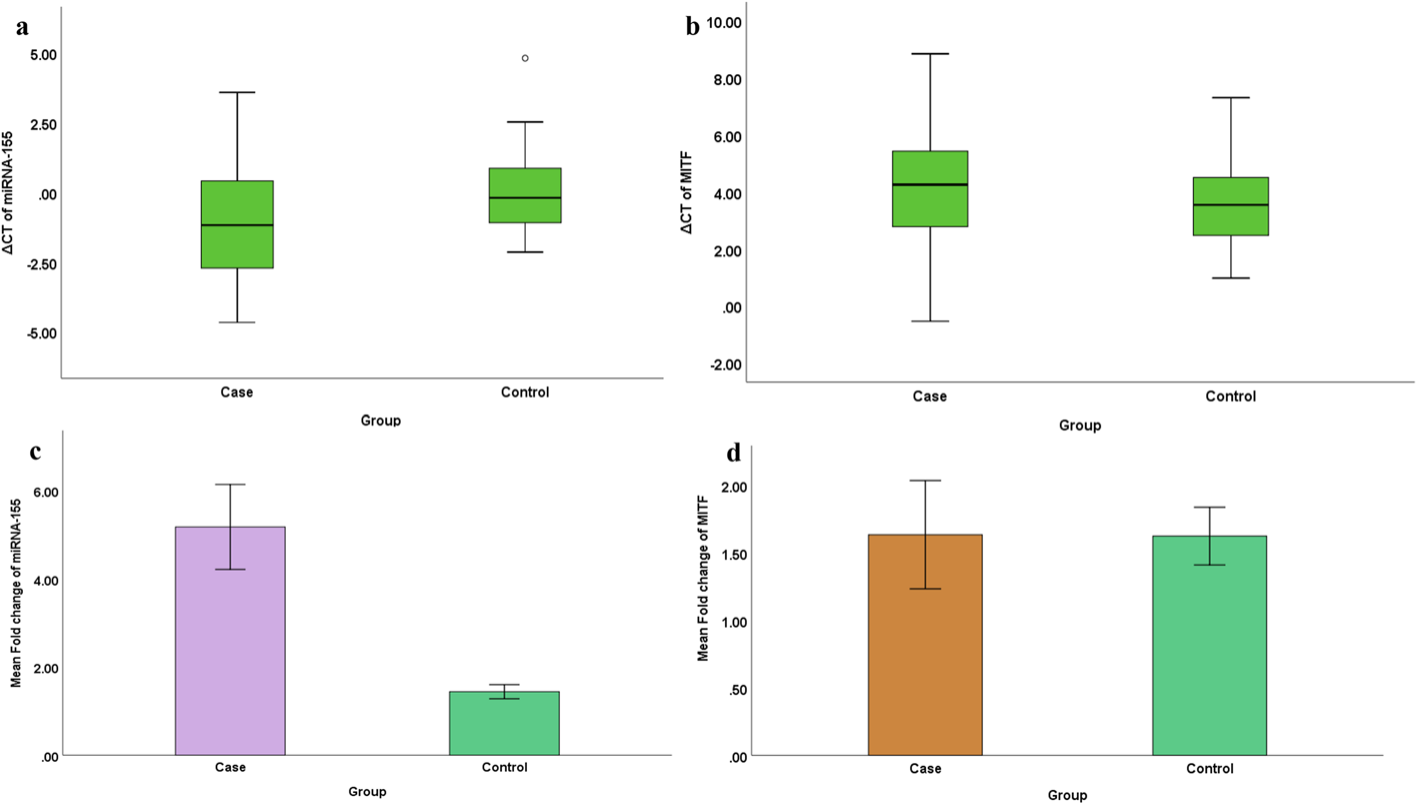
**a** Box and whisker plot showing ΔCT value of miRNA-155 in groups I and II. **b** Box and whisker plot showing ΔCT value of MITF gene expression in groups I and II. **c** Bar chart showing the mean fold change in miRNA-155 expression in groups I and II. **d** Bar chart showing the mean fold change in MITF gene expression in groups I and II

The mean ΔCT of miRNA-155 expression in groups I and II were −1.04 ± 2.26 and −0.01 ± 1.4 respectively. The mean ΔCT and fold change values of miRNA-155 expression showed a significant difference statistically between the groups (*P*≤0.01). A 5-fold increase in miRNA-155 expression was observed in the periodontitis group (*P*<0.01) (Table 3, Fig. 1).

**Table 3:** The mean standardized ΔCT and fold change values of miRNA-155 expression in groups I and II

Cyclic threshold (CT) value	Group I (mean ± SD)	Group II (mean ± SD)	Independent *t* test *P* Value
miRNA-155 ΔCT	−1.04 ±2.26	−0.01 ± 1.4	<0.01^**^
miRNA 155 Fold Change	5.18 ± 6.61	1.44± 1.12	<0.01^**^

^**^P value ≤0.01, statistically significant

The mean ΔCT of MITF gene expression in groups I and II were 4.15±2.16 and 3.51± 1.57 respectively. No significant difference in the fold change values of MITF gene expression was observed between the groups (*P*>0.01) (Table 4, Fig. 1).

**Table 4:** The mean standardized ΔCT and fold change values for MITF gene expression in groups I and II

Cyclic threshold (CT) value	Group I (mean ± SD)	Group II (mean ± SD)	Independent *t* test *P* value
MITF ΔCT	4.15± 2.16	3.51± 1.57	0.10, NS
MITF fold change	1.63 ±2.75	1.62± 1.49	0.98, NS

*P* value>0.01, *NS*- not statistically significant

### Correlation of site-specific clinical parameters with miR-155 and MITF gene expression in groups I and II and the total 98 samples (Table 5):

**Fig. 2:**
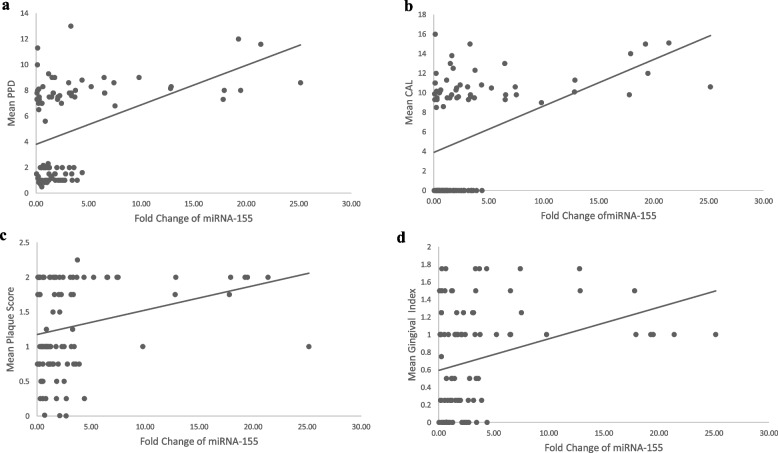
**a** Scatter plot showing the correlation between fold change of miRNA-155 with site-specific mean PPD in 98 subjects. **b** Scatter plot showing the correlation between fold change of miRNA-155 with site-specific mean CAL in 98 subjects. **c** Scatter plot showing the correlation between fold change of miRNA-155 with site-specific mean Plaque score in 98 subjects. **d** Scatter plot showing the correlation between fold change of miRNA-155 with site-specific mean Gingival Index score in 98 subjects

**Fig. 3:**
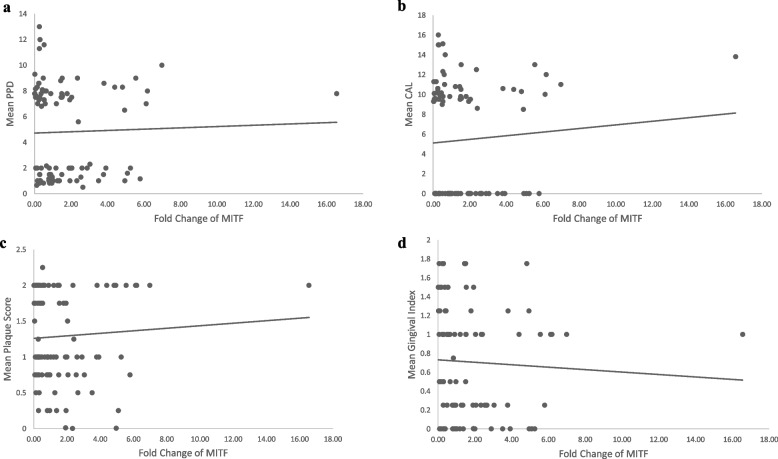
**a** Scatter plot showing the correlation between fold change of MITF gene expression with site-specific mean PPD in 98 subjects. **b** Scatter plot showing the correlation between fold change of MITF gene expression with site-specific mean CAL in 98 subjects. **c** Scatter plot showing the correlation between fold change of MITF gene expression with site-specific mean Plaque score in 98 subjects. **d** Scatter plot showing the correlation between fold change of MITF gene expression with site-specific mean Gingival Index Score in 98 subjects

In the total of 98 samples, the site-specific clinical parameters PPD, CAL, and GI showed a statistically significant strong negative and positive correlation with the ΔCT and fold change values of miRNA-155 expression, respectively (*P*<0.01). However, there was no correlation between MITF gene expression and site-specific clinical parameters (Figs. 2a–d and 3a–d). Similarly, no correlation was found between site-specific clinical parameters and ΔCT, fold change values of both the miRNA-155 and MITF gene expression in the groups I and II individually (Table and scatter plots included in the supplementary file).

**Table 5:** Spearman correlation between the standardized ΔCT and fold change values of miRNA-155 and MITF gene expression with the clinical parameters

		SS-PPD (mm)	SS-CAL (mm)	SS-PI	SS-GI
miRNA-155 ΔCT	*r*-value	−0.34^**^	−0.30^**^	−0.20	−0.25^**^
Fold change miRNA-155	*r*-value	0.34^**^	0.30^**^	0.20	0.25^**^
MITF ΔCT	*r*-value	0.07	0.07	0.03	0.16
Fold change MITF	*r*-value	−0.08	−0.09	−0.05	−0.20

*SS-PPD* site-specific mean probing pocket depth, *SS-CAL* site-specific mean clinical attachment loss, *SS-PI* site-specific Plaque index score, *SS-GI* site-specific Gingival Index score

^**^P value ≤0.01, statistically significant

### ROC analysis

**Fig. 4:**
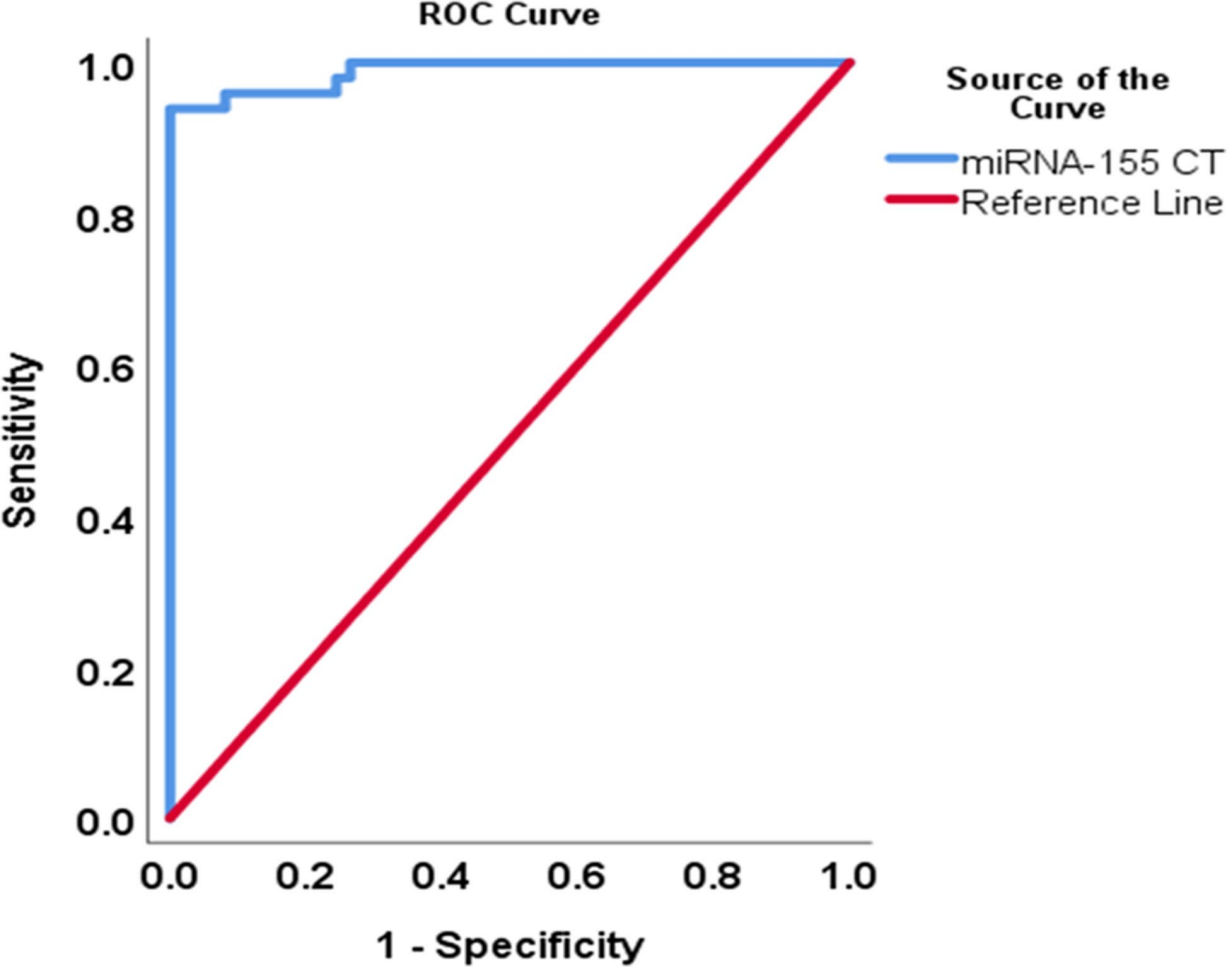
ROC curve discriminant analysis between periodontal health and disease based on miRNA-155 expression

ROC was used to evaluate the utility of the upregulated miR-155 expression to discriminate between periodontal health and disease and its potential to be used as a biomarker for periodontitis (Fig. 4). The analysis indicated that miRNA-155 showed high accuracy in discriminating periodontitis since the area under the curve (AUC) was 0.98 (95%CI: 0.97–1.0, SE = 0.008, *P* < 0.001) with a sensitivity of 93.8% (95%CI: 83.48–97.9), specificity of 100% (95%CI: 92.73–100), and diagnostic accuracy of 96.9% (95%CI: 91.38–98.95). The optimum cut-off value used to discriminate periodontitis from health was a standardized CT value ≥23.92. The positive predictive value was 100% (95%CI: 92.29–100) and the negative predictive value was 94.23% (95%CI: 84.36–98.02). MITF showed very low specificity (46.94%, 95% CI: 33.7–60.62) and 60.2% diagnostic accuracy to distinguish cases from controls (Table 6).

**Table 6:** AUC in ROC analysis based on the CT Values of miRNA-155 and MITF

Test result variable(s)	AUC	Std. error	*P*-value	Sensitivity (95% CI: LB, UB)	Specificity (95% CI: LB, UB)	Diagnostic accuracy (%)
miRNA-155 CT	0.98	0.01	0.00^**^	93.88% (83.48, 97.9)	100% (92.73, 100)	96.94%
MITF CT	0.56	0.05	0.27	73.47% (59.74, 83.79)	46.94% (33.7, 60.62)	60.2%

*AUC* area under the curve, *CI* confidence interval, *LB, UB* lower bound, upper bound

^**^P value ≤0.01, statistically significant

## Discussion

Dysregulated miRNAs have been reported in periodontitis. However, the data is limited and inconclusive, and the molecular mechanisms are complex and not yet thoroughly investigated. Therefore, we aimed to evaluate the expression of miRNA-155 in localized stage III/IV periodontitis and its regulation on MITF gene in the diseased periodontium. No studies till date have evaluated the regulation of osteoclastogenesis by miRNA-155 in periodontitis hence this research was undertaken.

In this study, miR-155 showed a fivefold increase in localized stage III/IV periodontitis when compared to periodontally healthy subjects (*P*<0.01). Similar findings have been reported by several authors [12–18]. The upregulated miRNA-155 exacerbates inflammation by inducing the gene expression of cytokines involved in inflammation via NF-kB signaling in the human gingival epithelial cells stimulated with periodontal pathogens [17]. The reduced levels of IL-10, a negative regulator of miRNA-155 in periodontitis, possibly could have contributed to the upregulation [19]. The upregulated miRNA-155 expression could be a protective response to periodontal inflammation or it could promote inflammation by encouraging macrophage differentiation and polarization toward the M1 phenotype [20]. However, future research on macrophage polarization and the markers for osteoclastogenesis including miRNA-155 expression may be needed to better understand the cardinal mechanisms leading to periodontal tissue destruction. The ROC analysis indicated that miRNA-155 could be an accurate biomarker for diagnosing periodontitis with an accuracy of 96.64% and this was in agreement with Wu et al. [13] and Al-Rawi et al. [14].

In contradiction, Xie et al. [21] observed a significantly reduced miR-155 expression in periodontitis and they attributed their inconsistent findings to the complex gingival environment and to the complicated processes involved in miRNA regulation. Nahid et al. [11] observed no significant changes in the miR-155 levels in the periodontium or spleen of experimental periodontitis in mouse model infected with *Porphyromonas gingivalis*, *Treponema denticola*, and *Tannerella forsythia.* Therefore, we can suggest that the ability of miRNA-155 to function as a pro- or anti-inflammatory factor is dependent on cell type, tissue of origin, microenvironment, stimuli, stimuli concentration, disease condition, and a host of other factors [22].

The MITF gene expression showed no difference between the diseased and healthy periodontium. Likewise, no difference was observed in the fold change between the groups (*P*>0.01). The MITF expression has not been studied in periodontitis and therefore could not be compared with any literature. It is possible that transcription factors other than MITF could dominate the process of osteoclast differentiation in periodontitis [23]. miR-155 had no effect on MITF gene expression in periodontitis in this study and this was completely contrary to the in vitro studies [6, 9, 20]. The discrepancy could be attributed to the in vitro parameters like culture medium, LPS type and dose, cell passage, and quantification methodologies that may not accurately reflect the in vivo environment. Moreover, a single gene can be regulated by multiple miRNAs; therefore, miRNAs other than miR-155 in the periodontal microenvironment could regulate MITF gene. Further, miRNA and gene expression profiles are tissue and cell specific; consequently, there will be differences in the expression depending on the disease and the environment being studied. Therefore, the established conclusions from in vitro and animal studies may not be applicable for in vivo circumstances and it is possible that MITF is not regulated by miRNA-155 in the periodontal milieu.

In the periodontitis group, no significant correlation was observed between the clinical parameters and the expression of miR-155 and MITF. On the contrary, correlating the site-specific clinical parameters of the total 98 samples showed a significant positive correlation with miRNA-155 fold change indicating its role in periodontal disease progression. Similar findings were reported by Wu et al. [13] and Motedayyen et al. [24].

Smaller sample size and cross-sectional study design are study limitations. Whole tissue specimens contain different cells in varying proportions between healthy and disease states, increased expression levels of a particular miRNA could be due to an increase in actual sequence transcription, an increase in the number of cell types harboring the transcript, or a combination of both. Isolating the cell type and analyzing cell-specific expression of a particular microRNA will, ideally, be able to discriminate between health and disease with accuracy. However, it would necessitate more advanced and expensive technology.

## Conclusion

Within the study limitations, it can be summarized that miRNA-155 expression was dysregulated and upregulated fivefolds in periodontal disease. Further, miRNA-155 could be used as a potential biomarker to discriminate between periodontal health and disease. The study findings indicated no difference in the MITF gene expression between periodontal health and disease. The results suggest that miRNA-155 did not affect the expression of MITF gene in the process of osteoclastogenesis in localized stage III/IV periodontitis.

## Supplementary Information

**Additional file 1.** Supplementary Figures and Tables.

## Data Availability

The data of the present study will be available on request. Supplementary file has been provided.

## Abbreviations

miRNA/miR: MicroRNA
MREs: miRNA response elements
mRNA: Messenger RNA
IL-1: Interleukin-1
IL-2: Interleukin-2
IL-4: Interleukin-4
IL-6: Interleukin-6
IL-11: Interleukin-11
IL-17: Interleukin-17
TNF-α: Tumor necrosis factor-α
PGEs: Prostaglandins
TGF-β: Transforming growth factor-β
IFN-γ: Interferon-γ
M-CSF: Macrophage colony-stimulating factor
MITF: Microphthalmia-induced transcription factor
ETS: Erythroblast transformation-specific
Erk: Extracellular signal-regulated pathway
PI3k: Phosphatidylinositol 3-kinase/protein kinase B
Akt: AK Strain Transforming
PI3k/Akt: Phosphatidylinositol 3-kinase/AK strain transforming
Bcl2: B cell lymphoma 2
NF-kB: Nuclear factor kappa-B
SOCS-1: Suppressor of cytokine signaling-1
MAPK: Mitogen-activated protein kinase pathway
qRT-PCR: Quantitative real-time polymerized chain reaction
TLR: Toll-like receptors
ROC: Receiver operating characteristic curve
AUC: Area under the curve
SS-PI: Site-specific Plaque index
SS-GI: Site-specific Gingival Index
BOP: Bleeding on probing
PPD: Probing pocket depth
CAL: Clinical attachment loss
FC: Fold change
CT: Cyclic threshold
ΔCT: Delta CT
ΔΔCT: Delta delta CT
OPG: Osteoprotegerin
BIC: B cell integration cluster
LPS: Lipopolysaccharides
GCF: Gingival crevicular fluid
M1: Macrophage phenotype 1
IRB: Institutional Review Board
EFP: European Federation of Periodontology
AAP: American Academy of Periodontology
