# Supplementary material for: Role of miRNA-155 in the regulation of osteoclast differentiation mediated by MITF in stage III/IV periodontitis: a case-control study
Source: J Genet Eng Biotechnol. 2022 Dec 2;20:161. doi: 10.1186/s43141-022-00441-1 (PMC9718899; doi:10.1186/s43141-022-00441-1)
Supplement: Supplementary file 1 — Additional file 1. Supplementary Figures and Tables. [file 43141_2022_441_MOESM1_ESM.docx]

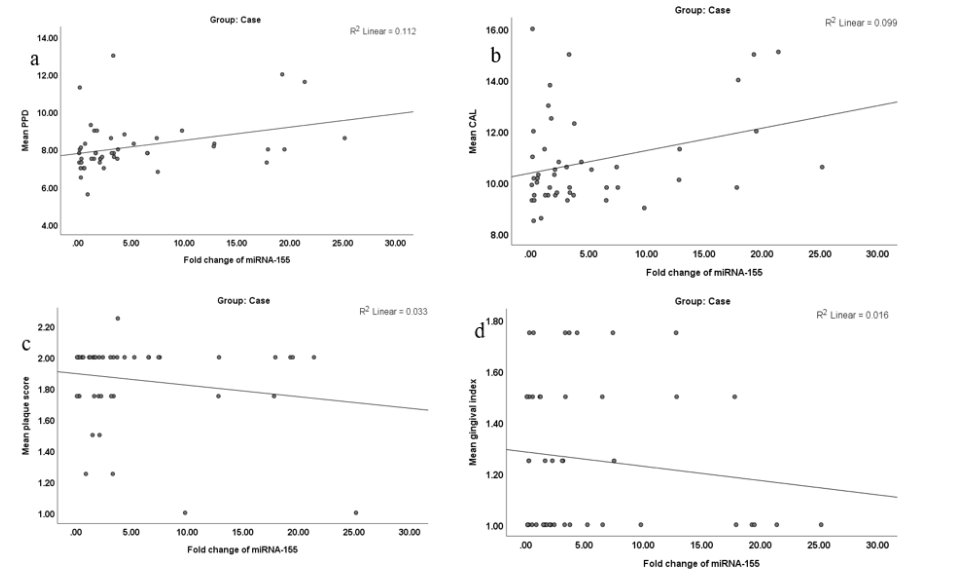


**Figure 1:**

a: Scatter plot showing the correlation between foldchange of miRNA-155 expression with Site Specific mean PPD in Group-I subjects.

b: Scatter plot showing the correlation between foldchange of miRNA-155 expression with Site Specific mean CAL in Group-I subjects.

c.Scatter plot showing the correlation between foldchange of miRNA-155 expression with Site Specific mean Plaque score in Group-I subjects.

d.Scatter plot showing the correlation between foldchange of miRNA-155 expression with Site Specific mean Gingival Index Score in Group-I subjects.


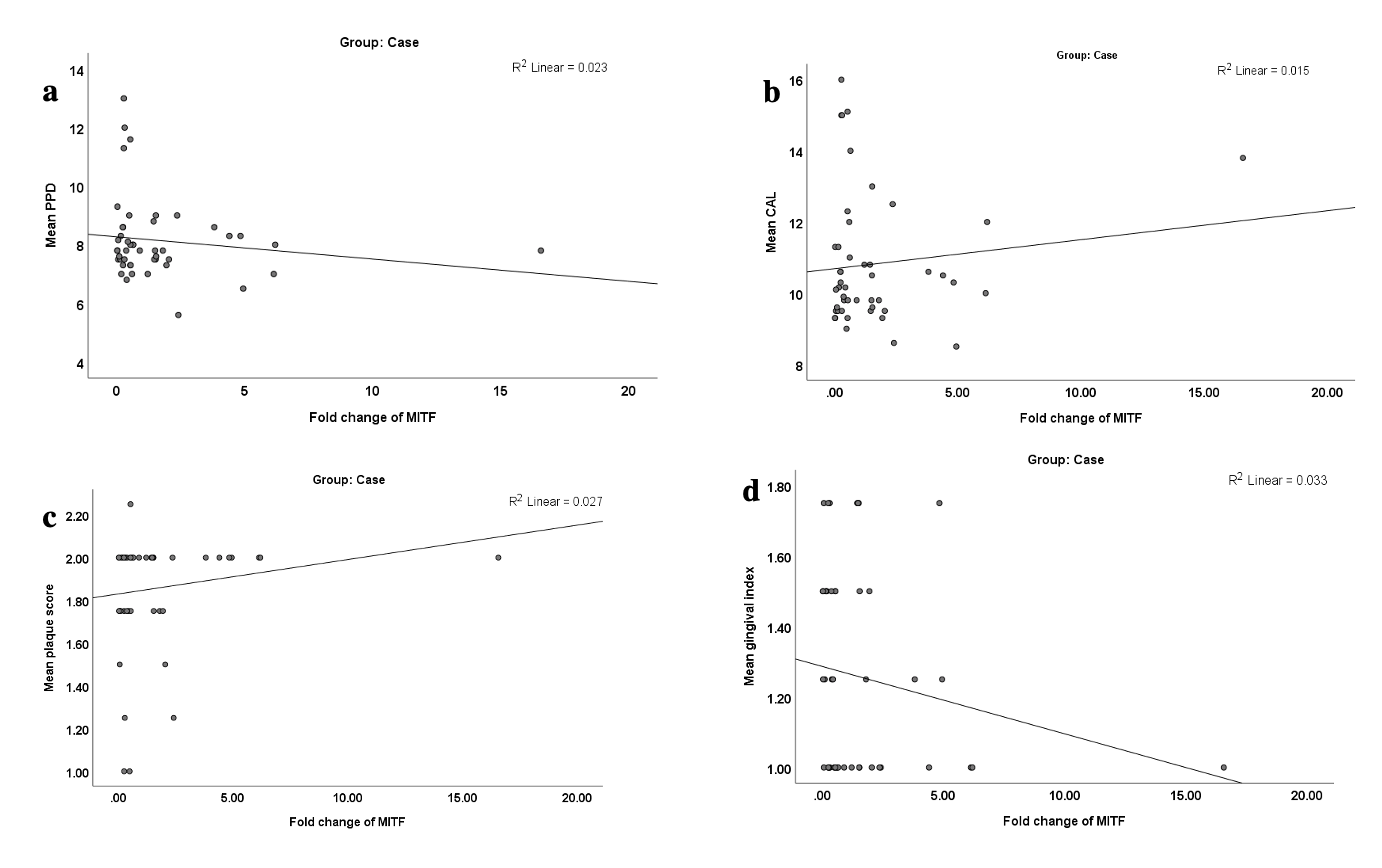


**Figure 2:**

a: Scatter plot showing the correlation between foldchange of MITF expression with Site Specific mean PPD in Group-I subjects.

b: Scatter plot showing the correlation between foldchange of MITF expression with Site Specific mean CAL in Group-I subjects.

c.Scatter plot showing the correlation between foldchange of MITF expression with Site Specific mean Plaque score in Group-I subjects.

d.Scatter plot showing the correlation between foldchange of MITF expression with Site Specific mean Gingival Index Score in Group-I subjects.


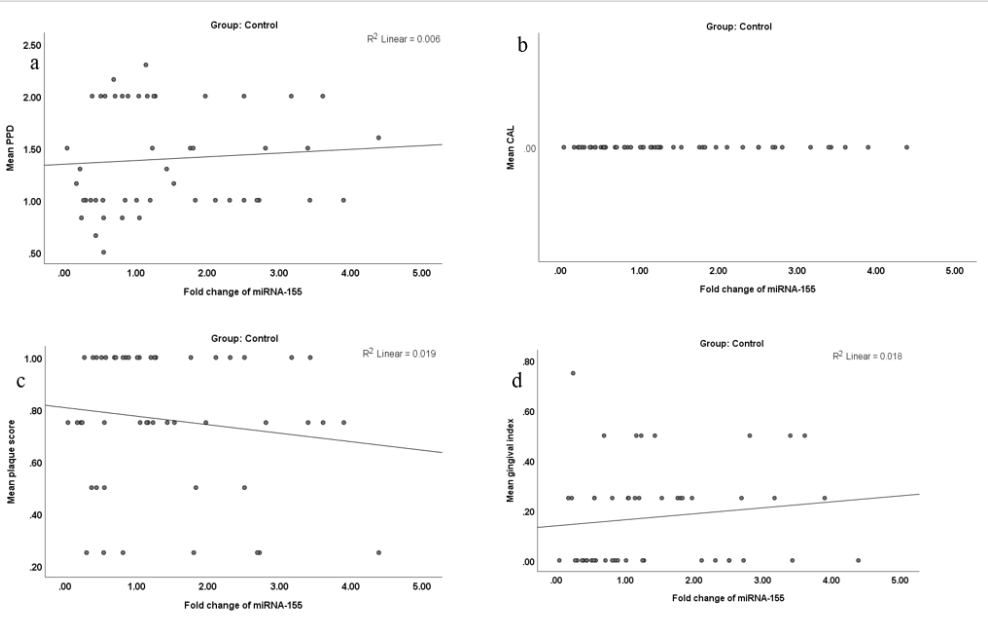


**Figure 3:**

a: Scatter plot showing the correlation between foldchange of miRNA-155 expression with Site Specific mean PPD in Group-II subjects.

b: Scatter plot showing the correlation between foldchange of miRNA-155 expression with Site Specific mean CAL in Group-II subjects.

c.Scatter plot showing the correlation between foldchange of miRNA-155 expression with Site Specific mean Plaque score in Group-II subjects.

d.Scatter plot showing the correlation between foldchange of miRNA-155 expression with Site Specific mean Gingival Index Score in Group-II subjects.


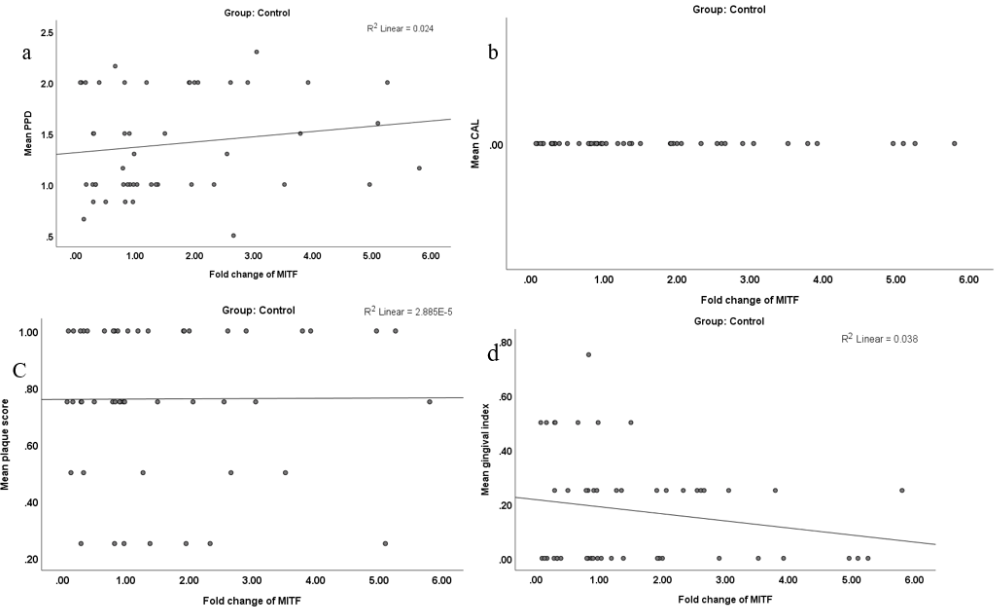


**Figure 4:**

a: Scatter plot showing the correlation between foldchange of MITF expression with Site Specific mean PPD in Group-II subjects.

b: Scatter plot showing the correlation between foldchange of MITF expression with Site Specific mean CAL in Group-II subjects.

c.Scatter plot showing the correlation between foldchange of MITF expression with Site Specific mean Plaque score in Group-II subjects.

d.Scatter plot showing the correlation between foldchange of MITF expression with Site Specific mean Gingival Index Score in Group-II subjects.

**Figure 5: Melting Curve analysis from qRT-PCR assay for miRNA-155 expression**

**
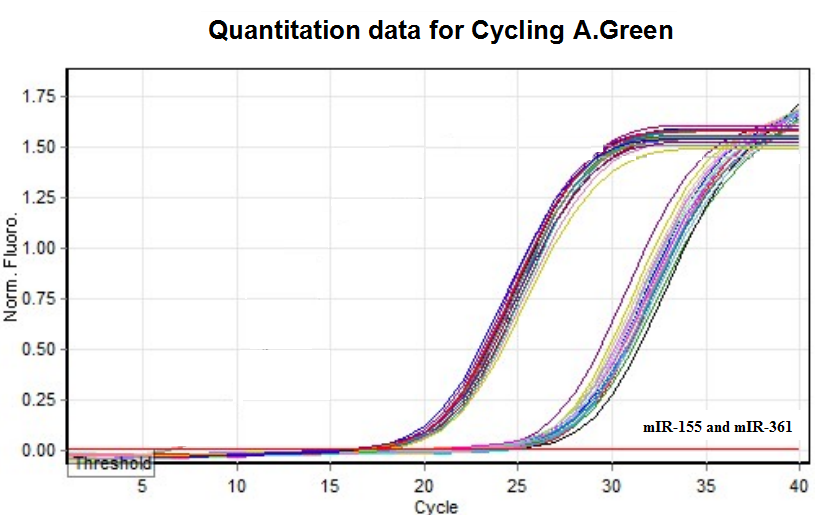
**

**Figure 6: Melting Curve analysis from qRT-PCR assay for MITF Gene expression**
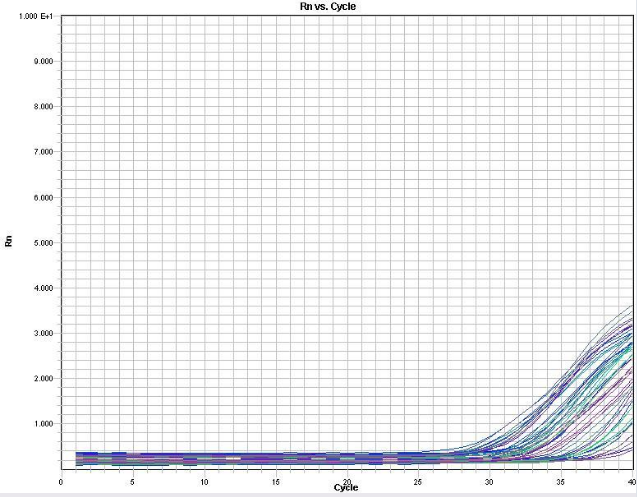


**Table 1:**

**Spearman correlation between the clinical parameters evaluated and ΔCT, foldchange values of miRNA-155 and MITF gene expression in Group I (Cases).**

|  | | Mean PPD | Mean CAL | Mean Plaque Index Score | Mean Gingival Index Score |
| --- | --- | --- | --- | --- | --- |
| miRNA-155 ΔCT | r-value | -0.30 | -0.20 | 0.03 | 0.05 |
| Fold change miRNA-155 | r-value | 0.30 | 0.19 | -0.02 | 0.05 |
| MITF ΔCT | r-value | 0.02 | -0.12 | -0.22 | 0.29 |
| Fold change MITF | r-value | -0.10 | 0.09 | 0.24 | -0.25 |

PPD- Probing Pocket Depth

CAL-Clinical Attachment Loss

MITF- Microphthalmia Induced Transcription Factor

**Table 2:**

**Spearman correlation between the clinical parameters evaluated and ΔCT, foldchange values of miRNA-155 and MITF gene expression in Group II (Controls).**

|  | | Site specific Mean PPD | Site specific Mean CAL | Mean Plaque Index Score | Mean Gingival Index Score |
| --- | --- | --- | --- | --- | --- |
| miRNA-155 ΔCT | r-value | -0.15 | -- | 0.05 | -0.22 |
| Fold change miRNA-155 | r-value | 0.15 | -- | -0.05 | 0.22 |
| MITF ΔCT | r-value | -0.17 | -- | -0.01 | 0.09 |
| Fold change MITF | r-value | 0.17 | -- | 0.01 | -0.10 |

PPD- Probing Pocket Depth

CAL-Clinical Attachment Loss

MITF- Microphthalmia Induced Transcription Factor
